# Advanced Signal Processing for Cardiovascular and Neurological Diseases

**DOI:** 10.1155/2018/3416540

**Published:** 2018-07-08

**Authors:** Dingchang Zheng, Fei Chen, Peng Li, Sheng-Yu Peng

**Affiliations:** ^1^Faculty of Medical Science, Anglia Ruskin University, Chelmsford CM1 1SQ, UK; ^2^Department of Electrical and Electronic Engineering, Southern University of Science and Technology, Shenzhen, China; ^3^Division of Sleep and Circadian Disorders, Brigham & Women's Hospital; Division of Sleep Medicine, Harvard Medical School, Boston, MA 02115, USA; ^4^Department of Electrical Engineering, National Taiwan University of Science and Technology, Taiwan

Advanced signal processing and computing techniques have been consistently playing a significant role in the field of biomedical engineering research. This special issue focused on the use and elaboration of latest advanced techniques for biomedical data analysis, including but not limited to deep machine learning, compressed sensing, and nonlinear dynamical approaches. Nine out of twenty-one submitted manuscripts in response to this special issue were finally accepted for publication, ranging from (i) noise suppression and removal in EEG and arterial photoplethysmography (PPG) signals; (ii) nonlinear dynamical approaches and multivariate and multiscale techniques for cardiovascular and neurophysiological imaging and signal processing; (iii) machine learning and deep neural network applications of cognitive outcome prediction for Alzheimer's diseases and Parkinson's diseases diagnosis; (iv) advanced signal processing to improve decision-making in brain-computer interface (BCI); and (v) acquisition and analysis of respiratory signals and rates using smartphones.

Since physiological signals are prone to measurement noise and capricious artefact, advanced signal processing is necessary to improve signal quality. W. Li et al. proposed a method based on independent component decomposition for common interference removal in a multichannel EEG recording system. W. Waugh et al. developed an algorithm to reduce sporadic noise in a continuous periodic signal, which has been validated in noise removal in arterial PPG signals. This technique can be generalised to be applicable to a wide range of other physiological signals. This special issue also accepted a study from Y. Chang and H. Wang who proposed a new channel compression technique in parallel MRI for imaging acceleration via kernel principal component analysis (KPCA).

Heart rate asymmetry (HRA) reflects the balancing regulation of the sympathetic and parasympathetic nervous systems. X. Wang et al. applied short-term HRA analysis to examine whether and how HRA changes during low intensity daily exercises. Once implemented into medical devices, this technique could be used for disease prediction to identify health-related alterations during daily life.

In the category of applying advanced biomedical signal analysis for diseases diagnosis and prediction, Z. Cai et al. developed an enhanced fuzzy k-nearest neighbor (FKNN) method by coupling the chaotic bacterial foraging optimization with Gauss mutation (CBFO) approach with FKNN for the early detection of Parkinson's disease. X. Liu et al. developed and evaluated linearized and kernelized sparse multitask learning for predicting cognitive outcomes in Alzheimer's disease. Their results showed that multitask learning methods not only achieved better prediction performance than the state-of-the-art competitive methods but also effectively fused the multimodality data.

Fast and reliable decision-making is important for real time BCI applications. R. Liu et al. applied the sequential probability ratio testing (SPRT) with power projective based method to speed up decision-making while trading off errors in BCI. O. Piña-Ramirez et al. proposed a "scenario" stimulation screen, which can be used for commanding a wheelchair even when users have no previous experience on the BCI spelling task.

Regarding the application of advanced signal processing in medical device development, this special issue accepted one study, which analysed cardiovascular signals utilizing mobile devices. L. Ge et al. designed and evaluated a smartphone-based respiration rate detection system utilizing single-frequency ultrasonic signals, where advanced respiration rate detection algorithm was also proposed.

In summary, we believe that this special issue has succeeded to collect papers covering many key areas of advanced signal processing for cardiovascular and neurological diseases. The special issue has provided an international forum for researchers working in the fields of biomedical engineering, medical physics, computational neuroscience, and integrative physiology to present the most recent ideas for understanding, diagnosing, and treatment of cardiovascular and neurological diseases. Ultimately, it will improve scientific debate in this interdisciplinary field.

